# PVT1 promotes proliferation and macrophage immunosuppressive polarization through STAT1 and CX3CL1 regulation in glioblastoma multiforme

**DOI:** 10.1111/cns.14566

**Published:** 2024-01-12

**Authors:** Lijie Huang, Zheng Wang, Chihyi Liao, Zheng Zhao, Hua Gao, Ruoyu Huang, Jing Chen, Fan Wu, Fan Zeng, Ying Zhang, Tao Jiang, Huimin Hu

**Affiliations:** ^1^ Department of Pathophysiology, Beijing Neurosurgical Institute Capital Medical University China; ^2^ Department of Neurosurgery, Beijing Tiantan Hospital Capital Medical University Beijing China; ^3^ Department of Molecular Neuropathology, Beijing Neurosurgical Institute Capital Medical University Beijing China; ^4^ Department of Cell Biology, Beijing Neurosurgical Institute Capital Medical University Beijing China; ^5^ Center of Brain Tumor Beijing Institute for Brain Disorders Beijing China; ^6^ China National Clinical Research Center for Neurological Diseases Beijing China; ^7^ Chinese Glioma Genome Atlas Network and Asian Glioma Genome Atlas Network Beijing China

**Keywords:** CX3CL1, DHX9, interferon‐stimulated genes, macrophages, PVT1

## Abstract

**Aims:**

This study aimed to investigate the role of plasmacytoma variant translocation 1 (PVT1), a long non‐coding RNA, in glioblastoma multiforme (GBM) and its impact on the tumor microenvironment (TME).

**Methods:**

We assessed aberrant PVT1 expression in glioma tissues and its impact on GBM cell growth in vitro and in vivo. Additionally, we investigated PVT1's role in influencing glioma‐associated macrophages. To understand PVT1's role in cell growth and the immunosuppressive TME, we performed a series of comprehensive experiments.

**Results:**

PVT1 was overexpressed in GBM due to copy number amplification, correlating with poor prognosis. Elevated PVT1 promoted GBM cell proliferation, while its downregulation inhibited growth in vitro and in vivo. PVT1 inhibited type I interferon‐stimulated genes (ISGs), with STAT1 as the central hub. PVT1 correlated with macrophage enrichment and regulated CX3CL1 expression, promoting recruitment and M2 phenotype polarization of macrophages. PVT1 localized to the cell nucleus and bound to DHX9, enriching at the promoter regions of STAT1 and CX3CL1, modulating ISGs and CX3CL1 expression.

**Conclusion:**

PVT1 plays a significant role in GBM, correlating with poor prognosis, promoting cell growth, and shaping an immunosuppressive TME via STAT1 and CX3CL1 regulation. Targeting PVT1 may hold therapeutic promise for GBM patients.

## Graphical abstract

PVT1 was upregulated in glioblastoma. It suppressed interferon signaling, altering immune cell infiltration. Additionally, PVT1 regulated CX3CL1, recruiting M2‐polarized glioma‐associated macrophages, and promoting an immunosuppressive microenvironment.

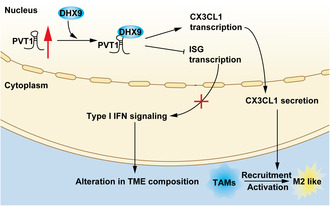

## 1 INTRODUCTION

Glioblastoma multiforme (GBM) is a highly malignant primary central nervous system (CNS) tumor. In recent two decades, there has been no groundbreaking progress in GBM treatment. The 5‐year survival rate of GBM is only 6.8%.^1^ The tumor microenvironment (TME) refers to the complex cellular and non‐cellular components surrounding tumor.^2^ In gliomas, the predominant immune cell population is macrophages, constituting 30%–50% of the tumor mass.^3^ Tumor‐associated macrophages (TAMs) in GBM microenvironment promote invasion and drug resistance in GBM by altering the tumor immune microenvironment.^2^ Therefore, a better understanding of the TAMs and its role in tumor progression might lead to the development of more effective cancer therapies.

In the GBM microenvironment, TAMs are composed of resident microglia and bone marrow‐derived macrophages (BMDMs).^3^ Multiple factors including MCP‐1, MCP‐3, hepatocyte growth factor, scatter factor, neurotrophic factors, CXCL12, CSF‐1, and GM‐CSF mediate the chemoattraction of TAMs to the GBM microenvironment.^4^, ^5^, ^6^, ^7^, ^8^, ^9^, ^10^ Macrophages and microglia exhibit various activation states. Distinct modes of macrophage activation had been delineated through in vitro stimulation. TAMs transform to the classic pro‐inflammatory M1 phenotype when exposed to Toll‐like receptor 4 (TLR4) ligands and IFN‐γ, whereas exposure to IL‐4, IL‐10, and IL‐13 drives TAMs turn toward an alternative M2 phenotype.^11^ TAMs polarization marker studies had revealed a predominance of M2‐like TAMs in gliomas.^12^ Glioma‐derived M‐CSF triggers transformation of microglia and macrophages toward the M2 phenotype and promotes tumor growth.^13^ Therefore, targeting pathways or molecules involved in the interaction between TAMs and gliomas presents a promising therapeutic strategy.

LncRNAs are a class of non‐coding RNA molecules that are longer than 200 nucleotides and do not encode proteins.^14^ Numerous lncRNAs play roles in shaping the TME and impacting tumor advancement. For instance, lncRNA IRENA converts chemotherapy‐polarized tumor‐suppressing macrophages to tumor‐promoting phenotypes in breast cancer.^15^ Similarly, CAFs promoted the metastatic activity of breast cancer cells by activating the transcription of lncRNA HOTAIR via TGF‐β1 secretion.^16^ Plasmacytoma variant translocation 1 (PVT1) is a long non‐coding RNA highly expressed in various types of tumors and has been associated with poor clinical outcomes.^17^, ^18^, ^19^, ^20^ PVT1 exhibits a strong association with the growth, invasiveness, epithelial–mesenchymal transition, and resistance to apoptosis, as well as a poor prognosis, and resistance to radiotherapy and chemotherapy.^21^, ^22^, ^23^, ^24^ However, there have been no reports about PVT1 impacts in GBM TME. In this study, we explore the involvement of PVT1 in the progression of GBM and its contribution to the development of an immunosuppressive microenvironment.

## 2 RESULTS

### 2.1 *PVT1* gene amplification in GBM and its expression correlate with molecular grading, serving as an indicator of poor prognosis in gliomas.

**FIGURE 1:**
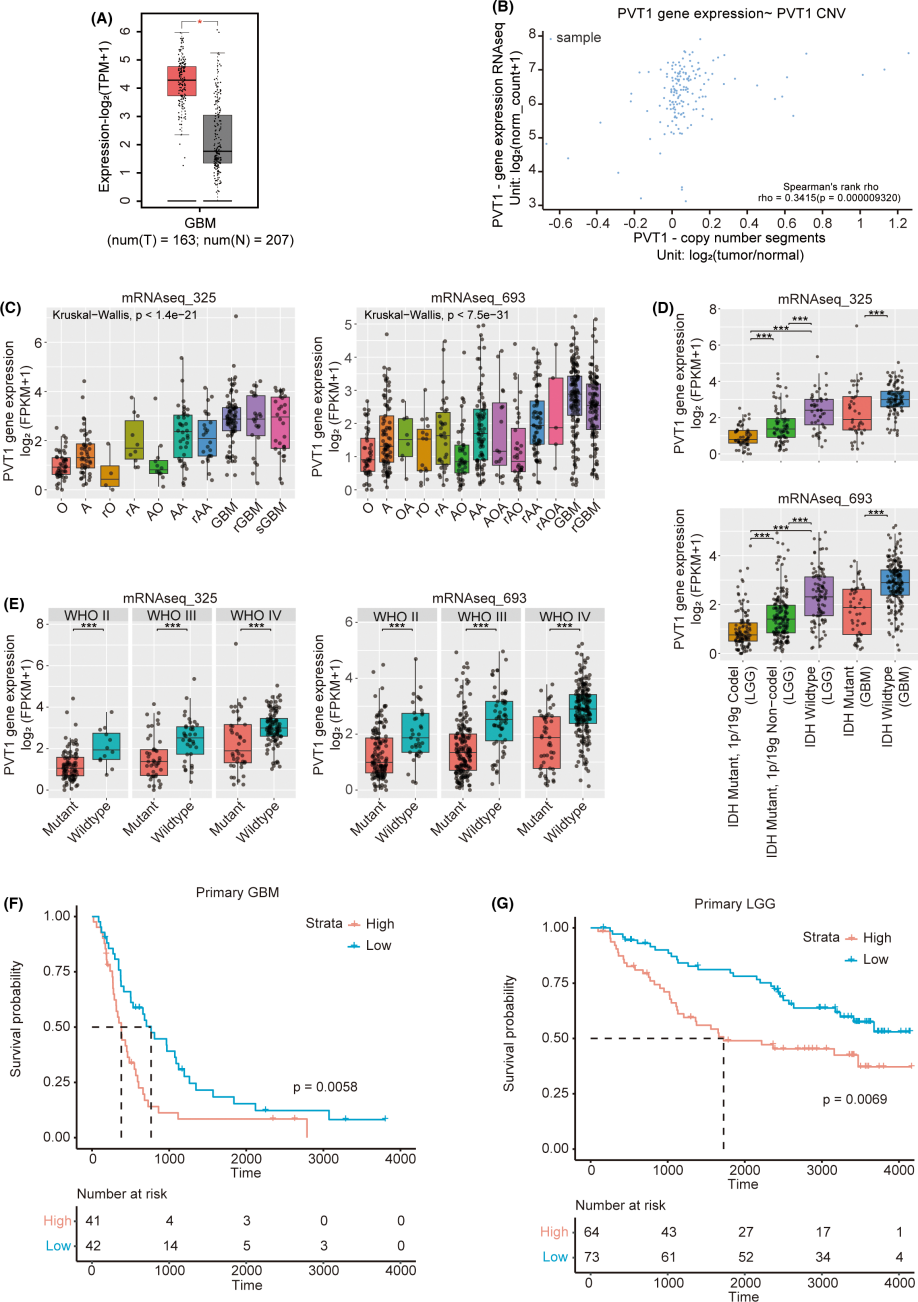
PVT1 is highly expressed in gliomas with poor prognosis. (A) The expression of PVT1 in GBM tissues and normal tissues is based on the GEPIA database (*p* < 0.05). (B) The correlation between the copy number of PVT1 and its expression was assessed using TCGA GBM data in UCSC Xena database. *R* = 0.3415 (*p* < 0.05). (C) The expression of PVT1 in glioma samples from CGGA database based on histopathological subtypes. Histological subtypes with less than five cases are not presented in the figure. (D) The expression of PVT1 in glioma samples from CGGA database according to the 2016 WHO molecular classification. (E) The expression of PVT1 in IDH‐mutant and wild‐type glioma samples from CGGA database. (F, G). The effect of PVT1 expression on the prognosis of patients with primary GBM and low‐grade gliomas in the CGGA database.

Through the analysis of the GEPIA database (http://gepia.cancer‐pku.cn),^25^ we observed higher expression of PVT1 in GBM tissues compared to normal tissues. *PVT1* gene is located on the chromosomal locus 8q24.21, a region frequently associated with copy number amplification in various cancer types.^26^, ^27^ To investigate whether the elevated expression of PVT1 in GBM is due to copy number amplification, we analyzed the correlation between copy number variations and gene expression in the TCGA GBM dataset using UCSC Xena (https://xenabrowser.net/).^28^ Our findings indicated a significant correlation between PVT1 copy number and its expression levels in GBM (Figure 1B).

Furthermore, by analyzing the Chinese Glioma Genome Atlas (CGGA)^29^ and TCGA glioma transcriptome sequencing data, we found a positive correlation between PVT1 expression levels and the 2016 WHO molecular grading of glioma, which increased with the rising pathological malignancy of glioma tissues (Figure 1C,D, and Figure S1A,B). Within the same grade of gliomas, the expression level of PVT1 in glioma of IDH wild type was notably higher than that in the ones of mutant type (Figure 1E and Figure S1C). These findings indicate a correlation between PVT1 expression and the molecular subtypes of gliomas. Kaplan–Meier analysis demonstrated that patients with high PVT1 expression had a significantly reduced overall survival (Figure 1F,G and Figure S1F,G).

### 2.2 PVT1 enhances GBM cell proliferation in vitro

**FIGURE 2:**
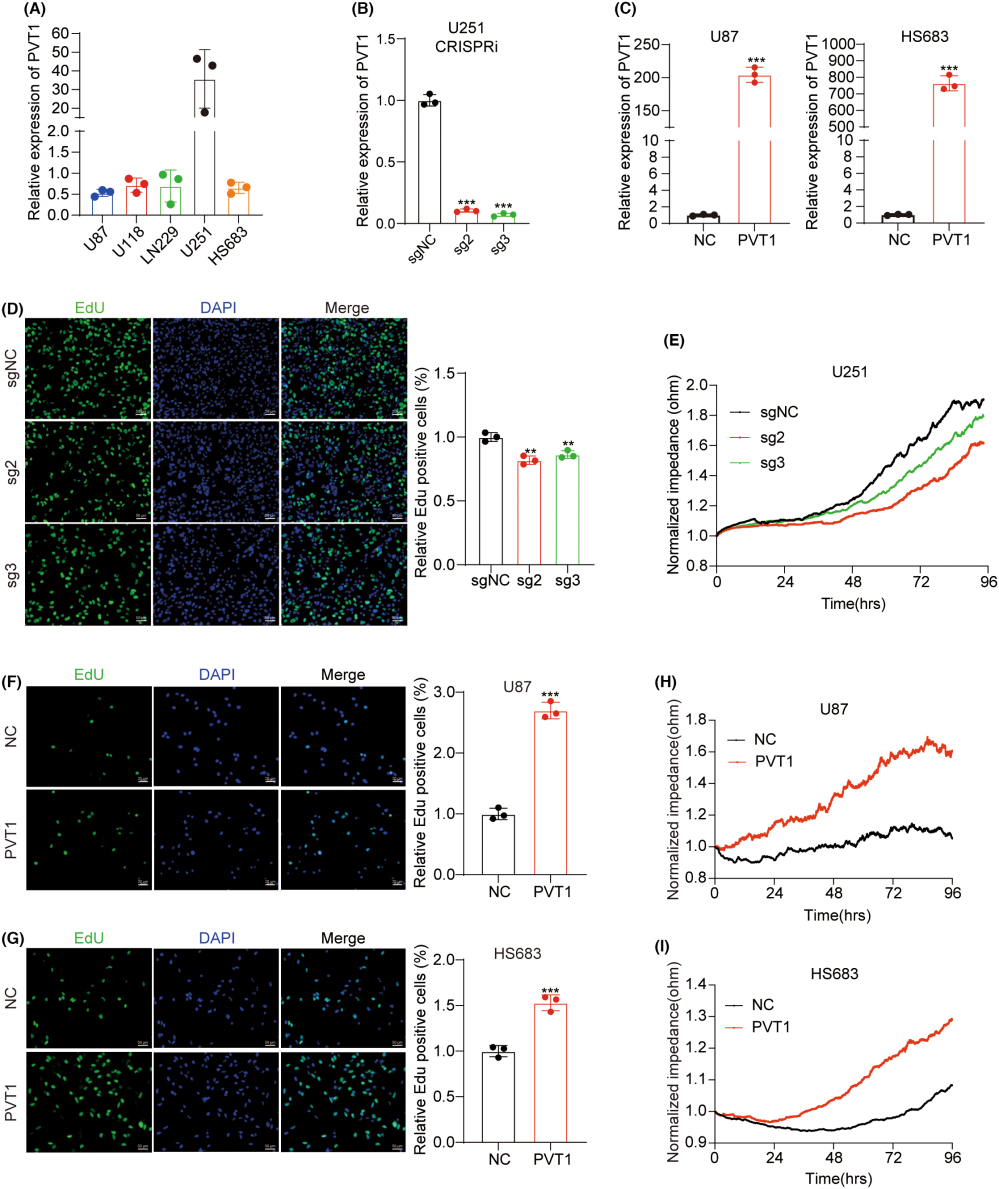
PVT1 enhances GBM cell proliferation in vitro. (A) The expression of PVT1 was detected in five GBM cell lines by RT‐qPCR. GAPDH was used as an internal reference. (B) RT‐qPCR analysis of PVT1 expression in U251 cells with negative control (sgNC) or PVT1‐specific sgRNAs (sg2/3). GAPDH was used as an internal reference. Statistical significance was assessed using one‐way ANOVA. ***p < 0.001. (C) RT‐qPCR analysis of PVT1 expression in stably PVT1‐expressing or control U87/HS683 cells. GAPDH was used as an internal reference. Statistical significance was assessed using unpaired Student's *t*‐test. ***p < 0.001. (D) EdU‐labeling experiment to assess the DNA replication activity in U251 cells with PVT1 stably knocked down. (E) The growth of U251 cells with PVT1 knockdown was monitored by the ECIS real‐time label‐free system. (F, G) EdU‐labeling experiment was conducted to assess the DNA replication activity in U87 and HS683 cells with stable high expression of PVT1. (H, I) The growth of U87 and HS683 cells with PVT1 overexpression was monitored using the ECIS real‐time label‐free system.

To further explore the function of PVT1 in GBM, we assessed the expression levels of PVT1 across multiple GBM cell lines (Figure 2A). U251 with PVT1 high expression was selected. We generated stable cell lines with reduced PVT1 expression using CRISPRi. Conversely, U87 and HS683 with PVT1 low expression were chosen to create stable cell lines with elevated PVT1 expression (Figure 2B,C). Subsequently, we conducted EdU‐labeling experiments to assess cellular DNA replication activity. Notably, the application of sgRNAs‐targeting PVT1 (sg2 and sg3) led to significant reduction in DNA replication activity (Figure 2D). Following this, we employed ECIS real‐time label‐free cell analysis to evaluate cellular proliferation capacity. The knockdown of PVT1 in U251 cells resulted in a remarkable decrease in proliferation (Figure 2E). Conversely, U87 and HS683 cells with elevated PVT1 expression exhibited a substantial increase in DNA replication activity (Figure 2F,G) as well as an enhancement in proliferation ability (Figure 2H,I). Collectively, the results of our in vitro experiments suggest that PVT1 acts as an oncogene in GBM.

### 2.3 PVT1 enhances GBM tumor growth in vivo

**FIGURE 3:**
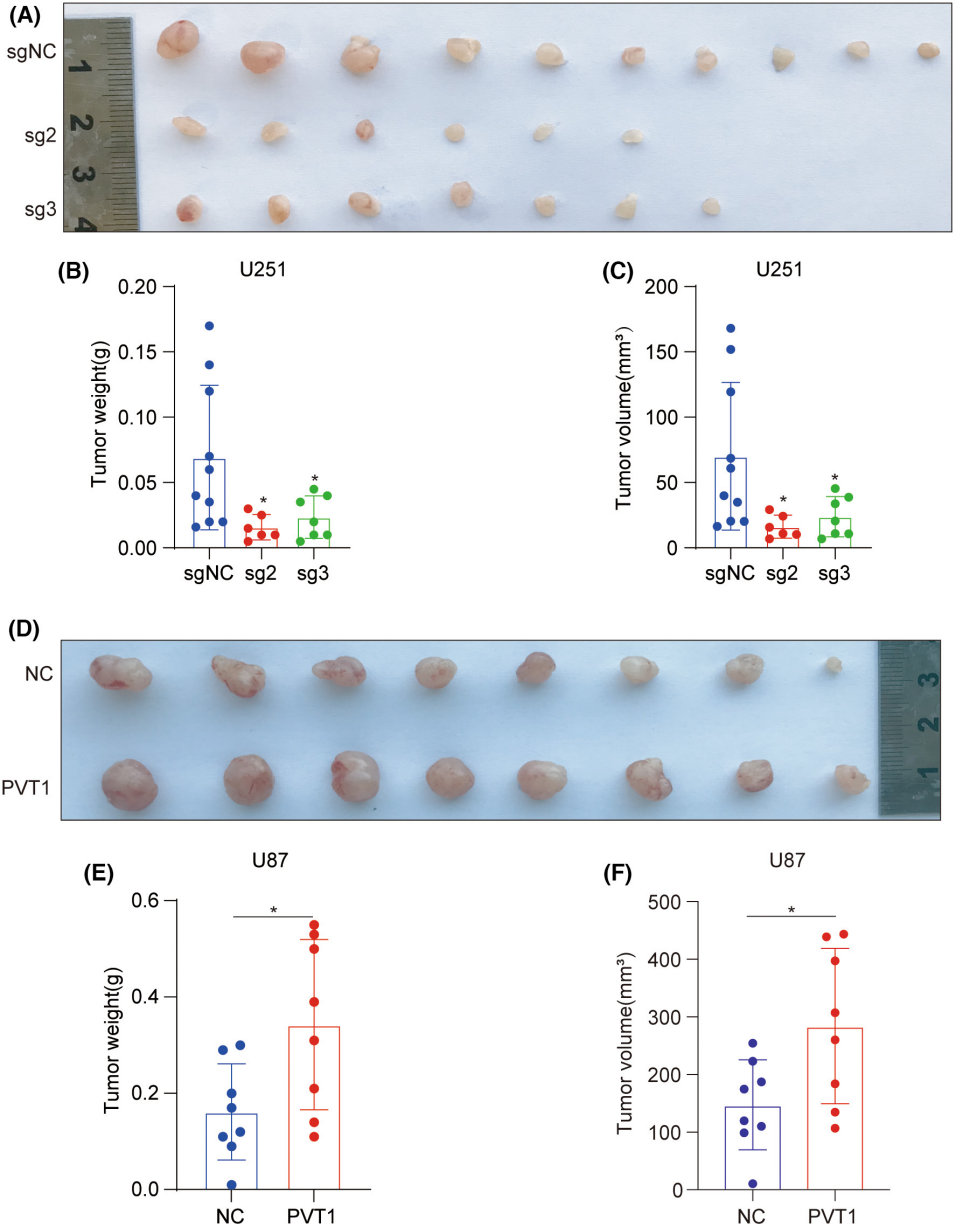
PVT1 enhances GBM cell proliferation in vivo. (A) Macroscopic appearance of xenograft tumors (1 month after transplantation) in nude mice following injection with U251 cells transfected by sgNC, sg2, or sg3 (*n* = 10). (B, C). Tumor weight and volume evaluation. Statistical significance was determined using one‐way ANOVA. *p < 0.05. (D) Macroscopic appearance of xenograft tumors (1 month after transplantation) in nude mice following injection with U87‐NC or U87‐PVT1 cells (*n* = 8). (E, F). Assessment of tumor weight and volume. Statistical significance was evaluated using Student's *t*‐test. *p < 0.05.

To further investigate the oncogenic role of PVT1 in GBM in vivo, we established a tumor xenograft model. U251 cells with PVT1 were stably knocked down and control cells were subcutaneously injected into the right shoulder of nude mice. We observed that the tumors had smaller volumes and lighter weights in the knockdown group, indicating a significant reduction in tumorigenic capacity due to compromised PVT1 expression (Figure 3A–C). Conversely, PVT1 overexpression enhanced the tumorigenic capability of U87 cells (Figure 3D,E). Therefore, these results collectively suggest that PVT1 promotes GBM tumor growth in vivo.

### 2.4 PVT1 suppresses the type I interferon receptor‐mediated signaling pathway and exhibits significant correlation with macrophage enrichment

**FIGURE 4:**
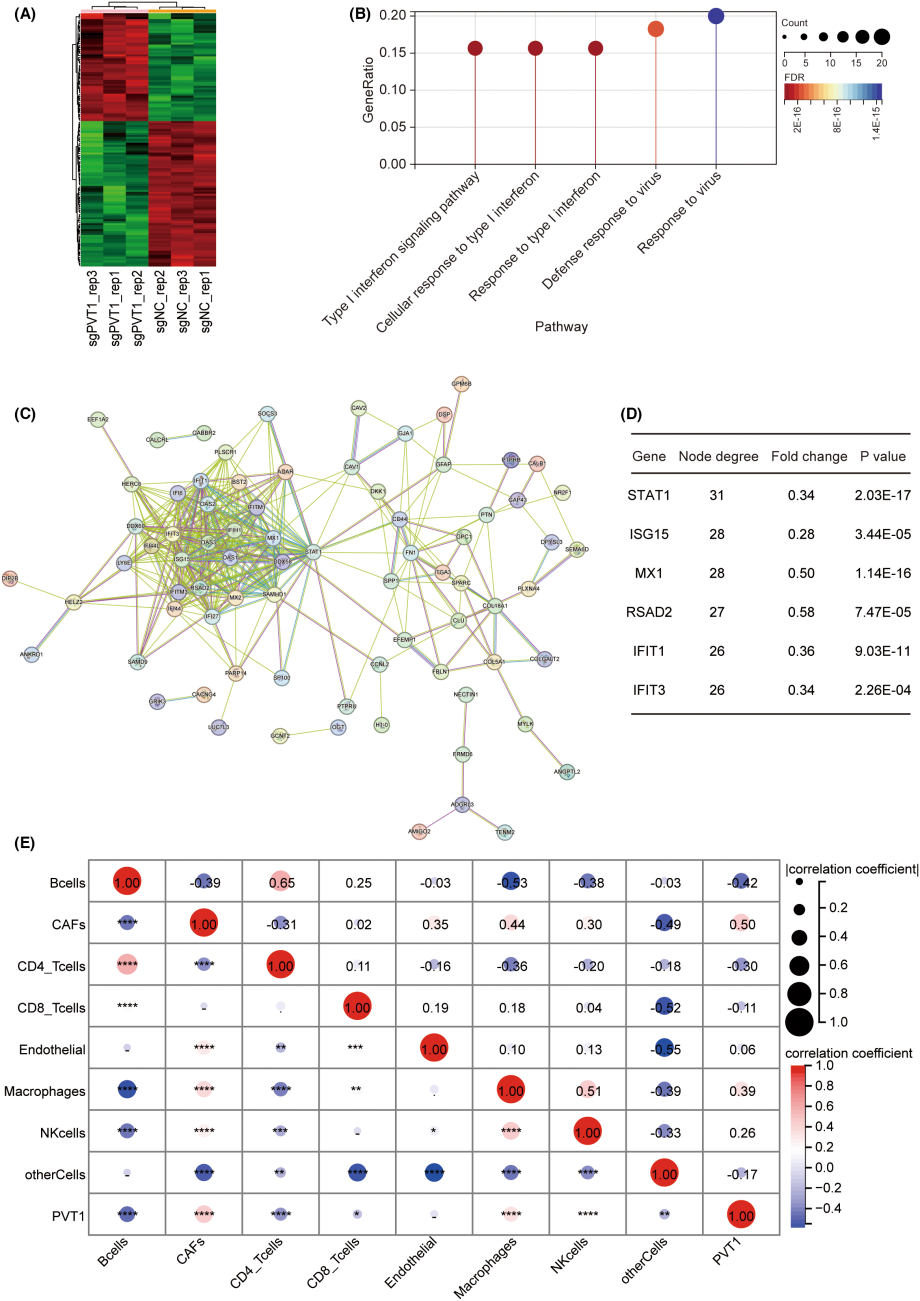
PVT1 suppresses the type I interferon receptor‐mediated signaling pathway and exhibits a significant correlation with macrophage enrichment. (A) Heatmap of the differentially expressed genes between U251 cells transfected with sgNC or sg3, according to RNA‐seq analysis. (B) Visualization of the top 5 most significant GO enrichment terms of the differentially expressed genes between U251 cells transfected with sgNC or sg3 (C) Protein–protein interaction (PPI) network of differentially expressed genes based on the STRING database. (D) The top 6 differentially expressed genes with the highest node degree scores in the PPI network. (E) Correlation between PVT1 expression and macrophage infiltration revealed through EPIC analysis of glioma tissues.

To further elucidate the role of PVT1 in GBM, we conducted transcriptome sequencing on U251 cells with PVT1 stably knocked down, employing a criterion of |log2 (Fold Change)| > 1 and *p*_adj_ < 0.05 to select differentially expressed genes (Figure 4A). Gene Ontology (GO) analysis of these differentially expressed genes revealed a significant enrichment of the interferon type I receptor‐mediated signaling pathway (Figure 4B). Furthermore, in order to elucidate the function of PVT1 in clinical samples, we intersected genes correlated with the expression of PVT1 from both CGGA and TCGA datasets (|*R*| ≥ 0.4). As a result, we identified 2347 genes correlated with PVT1. Subsequently, we subjected these genes to enrichment analysis. The results of the GO analysis demonstrated significant enrichment of the type I interferon signaling pathway (Figure S2A). Moreover, among the top 10 enriched pathways in the KEGG analysis, the majority were associated with viral infections, further emphasizing the impact of PVT1 on the type I interferon signaling pathway (Figure S2B). To identify the central genes regulated by PVT1, all differential genes were input into the STRING database to construct a protein–protein interaction (PPI) network (Figure 4C). STAT1, which had the highest score, was the hub gene of this network (Figure 4D). Considering that STAT pathway contributes to the conduction of interferon signaling,^30^ it is plausible that PVT1 might inhibit the interferon type I receptor‐mediated signaling pathway by downregulating STAT1.

Interferon type I plays a pivotal role in antitumor immunity.^31^, ^32^ To corroborate this, we performed EPIC (estimating the proportions of immune and cancer cells) analysis on RNA‐seq data from 325 cases of glioma tissues in the CGGA database. The significant correlation between PVT1 expression level and macrophage infiltration was revealed (Figure 4E). In summary, our findings indicate that PVT1 might affect tumor immune regulation via modulating the interferon type I receptor signaling pathway.

### 2.5 PVT1 promotes the recruitment and M2 phenotype polarization of macrophages via upregulating CX3CL1

**FIGURE 5:**
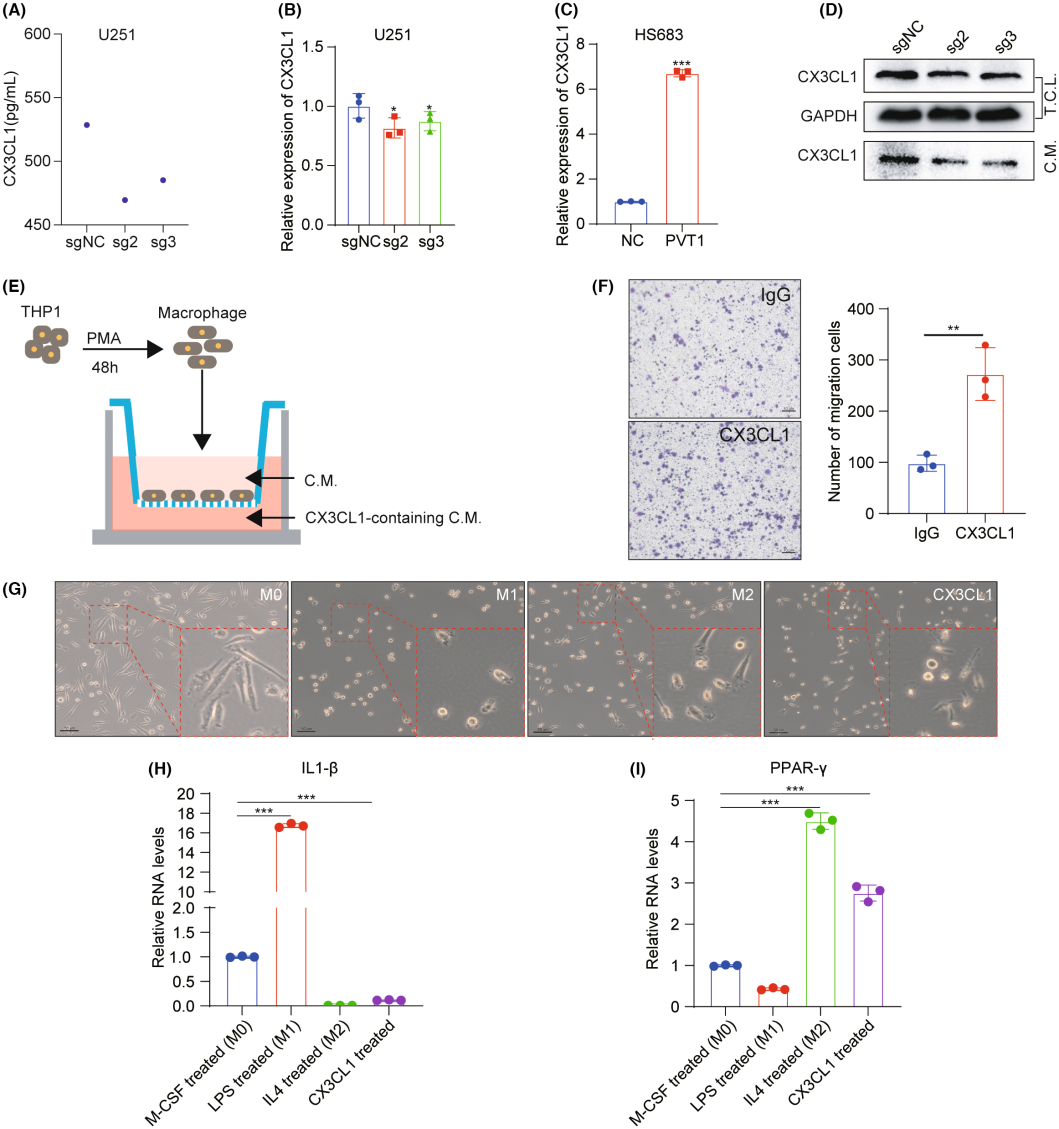
PVT1 fosters the recruitment of macrophages and facilitates their polarization toward the M2 phenotype via modulating CX3CL1 expression. (A) Chemokine array analysis revealed that downregulation of PVT1 led to a decrease in CX3CL1. (B) RT‐qPCR analysis of CX3CL1 expression in U251 cells with PVT1 knockdown. GAPDH was used as an internal reference. Statistical significance was assessed using one‐way ANOVA. *p < 0.05. (C) RT‐qPCR analysis of CX3CL1 expression in HS683 cells with PVT1 overexpression. GAPDH was used as an internal reference. Statistical significance was assessed using unpaired Student's *t*‐test. ***p < 0.001. (D) Western blot analysis of CX3CL1 protein level in total cell lysates (T.C.L.) or conditioned media (C.M.) of U251 cells with PVT1 knockdown. (E) The schematic diagram of transwell experiments designed to evaluate CX3CL1's capacity to recruit macrophages. (F) The chemotactic abilities of THP1 cells were assessed using a transwell assay. Representative images (left) and quantitative analyses (right) are shown. Scale bars: 10 μm. Statistical significance was determined using an unpaired Student's *t*‐test. **p < 0.01. (G) Micrographs of macrophages treated with 100 ng/mL of LPS, 20 ng/mL of IL‐4, or 200 ng/mL of CX3CL1 for 48 h. LPS induced macrophage polarization toward the M1 phenotype, and IL4 promoted macrophage polarization into the M2 phenotype. Scale bars: 50 μm. (H, I) RT‐qPCR analysis of IL1‐β and PPAR‐γ in macrophages following treatment with LPS, IL‐4, or CX3CL1. GAPDH was used as an internal reference. Statistical significance was assessed using one‐way ANOVA. ***p < 0.001. IL1‐β serves as a marker for M1 phenotype macrophages, while PPAR‐γ is indicator of M2 phenotype macrophages.

To elucidate the relationship between PVT1 expression and macrophage enrichment, we collected conditioned medium of cells with stable PVT1 knockdown. Chemokine array revealed a significant decrease in CX3CL1 in these conditioned media (Figure 5A). In cells with PVT1, stably knocked‐down mRNA and protein expression of CX3CL1 were decreased (Figure 5B,D). Conversely, elevating PVT1 expression resulted in an increased mRNA level of CX3CL1 (Figure 5C). These findings collectively suggest a potential role of PVT1 in transcriptionally regulating CX3CL1 expression.

The receptors for CX3CL1 are predominantly expressed in monocytes.^33^, ^34^ Hence, we conducted transwell experiments to investigate the potential of CX3CL1 to recruit macrophages. Initially, human monocytic cell‐line THP1 was induced into macrophages using PMA. Subsequently, the macrophages were seeded in the upper chamber, while the lower chamber was filled with culture medium containing CX3CL1 (Figure 5E). The results demonstrated that CX3CL1 was effective in enhancing macrophage chemotaxis (Figure 5F).

To investigate whether CX3CL1 influences the polarization state of macrophages, we initially activated human peripheral blood mononuclear cells into macrophages using M‐CSF. Subsequently, we treated the macrophages with culture media containing LPS, IL4, or CX3CL1, respectively. The outcomes demonstrated that LPS induced macrophage polarization toward the M1 phenotype. IL4 promoted macrophage polarization into the M2 phenotype (Figure 5H,I), while macrophages treated with CX3CL1 exhibited a morphology more closely to the M2 phenotype (Figure 5G). Additionally, after CX3CL1 treatment, the M2‐polarized macrophage marker PPAR‐γ was upregulated, while the M1‐polarized macrophage marker IL1‐β exhibited a decrease (Figure 5H,I). These findings collectively suggest that CX3CL1 facilitates the polarization of macrophages toward M2 phenotype.

### 2.6 Direct interaction of PVT1 with DHX9 modulates expression of type I interferon‐stimulated genes and CX3CL1

**FIGURE 6:**
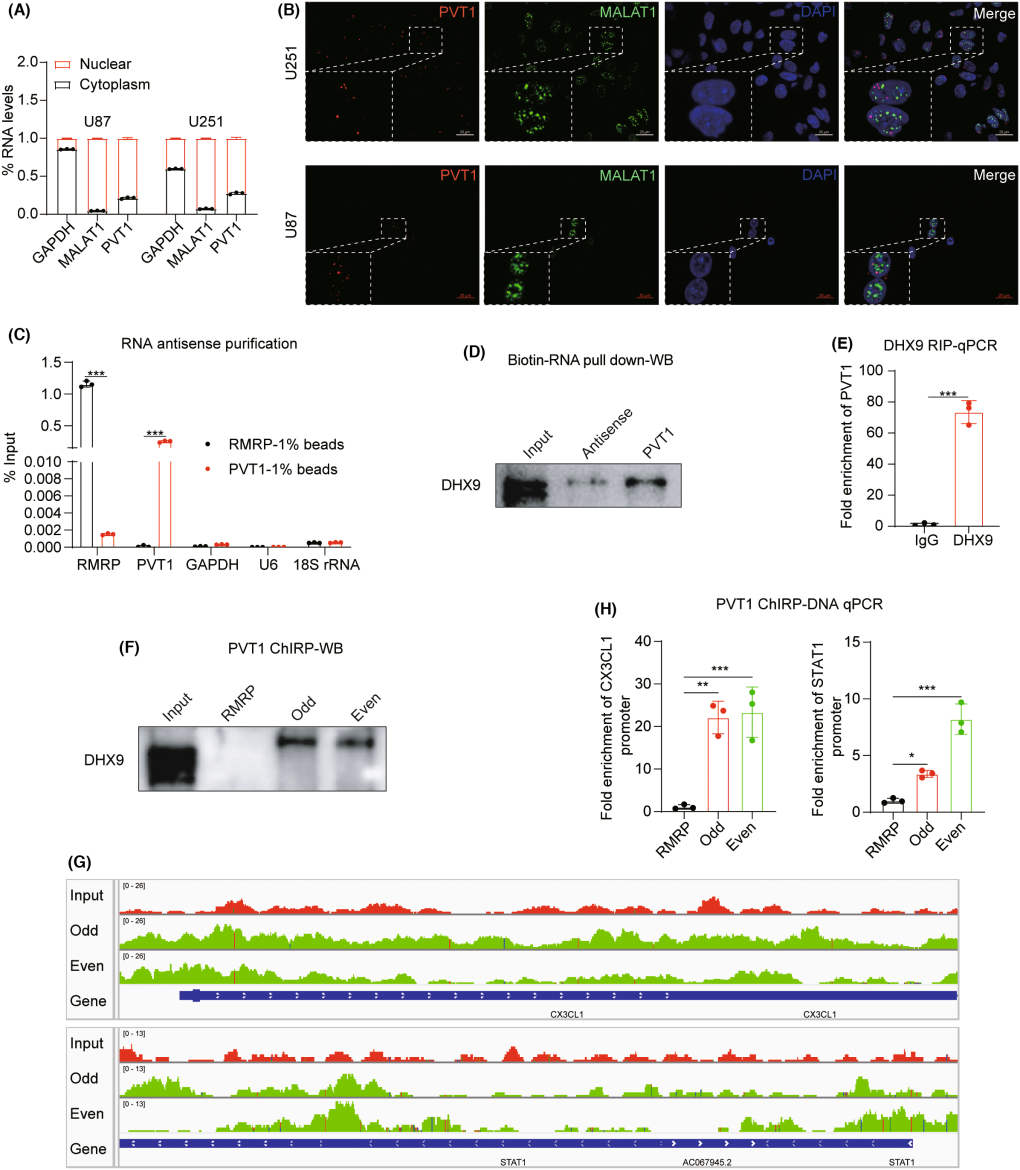
Direct interaction of PVT1 with DHX9 modulates expression of type I interferon‐stimulated genes and CX3CL1. (A) RT‐qPCR analysis of PVT1 expression in nucleus or cytoplasm of U87 and U251 cells. GAPDH was used as the cytoplasmic control transcript, and MALAT1 was used as a nuclear RNA control. (B) Stellaris RNA FISH with PVT1 (red) and MALAT1 (green) probes showing the subcellular location of PVT1 in U251 cells. Scale bars: 20 μm. (C) RT‐qPCR analysis was conducted to assess the specificity of PVT1 and RMRP probes in RAP experiments. GAPDH, U6, and 18S rRNA were employed as negative controls to demonstrate the specific enrichment of target RNA by PVT1 and RMRP probes. Statistical significance was assessed using two‐way ANOVA. ***p < 0.001. (D) Western blotting validation of the interaction between PVT1 and DHX9 following RNA pull‐down assay. (E) RIP coupled RT‐qPCR assays detecting the interaction between DHX9 and PVT1 in U251 cells. The enrichment of PVT1 coupled with anti‐DHX9 antibody was compared to IgG control. Statistical significance was assessed using an unpaired Student's *t*‐test. ***p < 0.001. (F) The western blotting results of chromatin isolation by RNA purification (ChIRP) showed that PVT1 binds to DHX9. The PVT1 probes are divided into odd and even groups. (G) The results of ChIRP‐seq showed that PVT1 bound to STAT1 and CX3CL1 promoter. The integrative genomics viewer (IGV) was used to visualize the results of ChIRP‐seq. (H) PVT1 ChIRP coupled RT‐qPCR assays to detect the enrichment of the STAT1 and CX3CL1 promoter regions. The enrichment of promoter region of CX3CL1 and STAT1 was compared to RMRP control. Statistical significance was assessed using one‐way ANOVA. *p < 0.05, **p < 0.01, and ***p < 0.001.

In order to elucidate the mechanism underlying PVT1's function, subcellular fractionation experiments were employed to determine the nuclear and cytoplasmic distribution of PVT1. The results revealed predominant nuclear localization of PVT1 in U87 and U251 cells (Figure 6A). This observation was further substantiated by Stellaris RNA fluorescent in situ hybridization (FISH) (Figure 6B).

Subsequently, we employed RNA antisense purification–mass spectrometry (RAP‐MS) to identify protein molecules directly binding to PVT1 within live cells. By incorporating 4‐thiouridine into U251 cells, covalent cross‐linking of proteins and RNA was achieved under UV irradiation. Biotin‐labeled PVT1 probes were employed to capture the PVT1 RNA–protein complex. Purified RNA molecules were validated using RT‐qPCR, while purified protein molecules underwent mass spectrometry analysis. To demonstrate the specificity of the probe, an additional set of control probes targeting the RNA component of mitochondrial RNA‐processing endoribonuclease (RMRP), another long non‐coding RNA, were designed.^35^ RAP results revealed that the PVT1 probes exclusively bound to PVT1 RNA, while the RMRP probe exclusively bound to RMRP RNA. Notably, both probe sets exhibited minimal binding to highly abundant cellular RNA like GAPDH, U6, and 18S rRNA. This unequivocally indicates that the PVT1 probe can selectively bind to the PVT1 RNA–protein complex (Figure 6C). Subsequently, the proteins bound by the PVT1 probes were subjected to mass spectrometry analysis, with DHX9 emerging as one of the top‐ranking proteins. Furthermore, the direct interaction of PVT1 and DHX9 was further validated through bio‐RNA pull‐down experiments (Figure 6D). Additionally, RNA immunoprecipitation (RIP) experiments corroborated that DHX9 can indeed bind to PVT1 (Figure 6E).

Given that PVT1 localizes within cell nucleus, we hypothesized that PVT1 and DHX9 might be involved in the transcriptional regulation of downstream genes. Chromatin isolation by RNA purification (CHIRP) is a technique utilized to study the interactions among RNA, DNA, and proteins. In our investigation, we employed PVT1 CHIRP to study the DNA chromosome fragments by PVT1 binding, as well as the RNA‐binding proteins involved in transcriptional regulation. CHIRP‐western blot results convincingly demonstrated the specific binding of both odd and even sets of PVT1 probes to DHX9 (Figure 6F). CHIRP‐DNA‐seq and CHIRP‐DNA‐qPCR indicated significant enrichment of the promoter regions of CX3CL1 and STAT1 within the PVT1 probe sets (Figure 6G,H). Collectively, these findings substantiate a direct interaction between PVT1 and DHX9, contributing to transcriptional regulation that influences the expression of genes associated with the interferon type I pathway and the chemokine CX3CL1.

## 3 DISCUSSION

LncRNAs have been established as key regulators of glioma hallmarks, including sustained proliferation,^36^ resistance to cell death,^37^ and induction of angiogenesis.^38^ Despite these significant findings, the precise regulatory roles of lncRNAs in glioma progression and TME have yet to be fully elucidated. In this study, we observed high expression of PVT1 in GBM. *PVT1* gene is located in a genomic region frequently subject to copy number amplification in tumors. The proto‐oncogene MYC is the most functionally significant oncogene in this genomic region.^26^, ^27^ A study investigated copy number variations in medulloblastoma identified translocation of PVT1 as a novel molecular subtype.^39^ In pediatric midline gliomas and astrocytomas with K27M mutations, MYC/PVT1 also exhibited copy number amplification and patients with this subtype experienced a poorer prognosis.^40^, ^41^ PVT1 has been reported to facilitate proliferation and migration of GBM by acting as a miRNA sponge.^42^ Here, we observed high expression of PVT1 due to copy number amplification in GBM. Furthermore, we found the expression level of PVT1 correlated with molecular grading, serving as an indicator of poor prognosis in gliomas. Based on cellular and animal experiments, PVT1 could promote GBM cell proliferation in vitro and in vivo.

PVT1 suppressed the expression of type I interferon‐stimulated genes (ISGs), including STAT1, MX1/2, ISG15, OAS1/2/3, etc. Among them, STAT1 serves as the hub gene in the PPI network formed by PVT1‐related DEGs (differentially expressed genes). Considering that interferon (IFN) signaling predominantly triggers the transcriptional activation of ISGs via STAT pathway,^30^ it is plausible that PVT1 might inhibit the interferon type I receptor‐mediated signaling pathway by downregulating STAT1.

In addition to antiviral properties, type I IFNs play a significant role in antitumor immunity. They facilitate apoptosis of tumor cells, suppress proliferation, regulate differentiation, and enhance MHC‐mediated antigen presentation.^31^, ^32^ Type I IFNs also have a crucial impact on antitumor immune responses in GBM. In an animal model study, it was observed that CD4^+^ T cells, which inhibit tumor progression, promote angiogenesis and carcinogenesis in the absence of TNFR1 or IFN‐signaling stimulation.^43^ In glioblastoma animal models with the type I IFN receptor knocked out (Ifnar1−/−), tumor growth is accelerated, and mortality occurs earlier.^44^ Therefore, PVT1‐mediated inhibition of the type I IFNs signaling potentially modify the components of GBM microenvironment. Actually, we confirmed the significant correlation between PVT1 expression and macrophage infiltration.

In healthy brain, the CX3CL1 receptor CX3CR1 is primarily expressed by microglial cells and has been utilized as a marker for microglial cell imaging.^33^ However, other studies have demonstrated that blood monocytes also express CX3CR1, whose level was upregulated during monocyte differentiation into macrophages. In this study, we discovered that CX3CL1 promotes macrophage recruitment and facilitates the polarization toward the M2 phenotype.

Mechanistically, we demonstrated that PVT1 could specifically interact with DHX9. DHX9, also known as RNA helicase A (RHA) or nuclear DNA helicase II (NDHII), is classified as an NTP‐dependent RNA/DNA helicase. This enzyme exhibits both RNA and DNA unwinding activities. In the realm of transcriptional regulation, DHX9 plays a vital role as a coactivator, capable of directly binding to promoters or acting as a mediator between transcription factors and co‐factors.^45^ We elucidated PVT1 and DHX9 enrich at the promoter regions and regulate the expressions of STAT1 and CX3CL1. Collectively, our study proposed that PVT1 is a promising therapeutic target to disrupt the immunosuppressive microenvironment and combat GBM progression.

## 4 MATERIALS AND METHODS

### 4.1 Cell culture

U87, U251, HS683, and THP1 cell lines were obtained from the National Biomedical Experimental Cell Resource Bank, while CD14^+^ monocytes were purchased from AllCells Inc. U118 and LN229 cell lines were previously maintained in our laboratory. U87, HS683, U251, U118, and LN229 cells were cultured in DMEM (Gibco) supplemented with 10% fetal bovine serum (Gibco). THP1 cells and CD14^+^ monocytes were cultured in RPMI 1640 (Gibco) medium with 10% FBS. All the cells were maintained at 37°C in a humidified atmosphere containing 5% CO_2._

### 4.2 Establishment of stable cell lines expressing PVT1

Stable high‐expression PVT1 cell lines, including U87‐PVT1, HS683‐PVT1, were achieved through lentivirus‐mediated infection of U87 and HS683 cells, followed by selection with 2 μg/mL puromycin. The lentiviral particles were packaged by Genechem Inc. (China).

We generated stable knockdown of PVT1 in U251 cells using CRISPR interference (CRISPRi). Lentiviral vectors employed in this study were plenti‐CMV‐dspCas9‐KRAB‐2A‐puro and plenti‐U6‐gRNA‐2X(wt + f6)‐MS2‐CMV‐NLS‐MCP‐KRAB‐IRES‐blasticidin. The sequences of the sgRNAs can be found in Table S1. Lentiviral particles were packaged by SHBIO Biotechnology (China). These cells were then selected in a medium supplemented with 2 μg/mL puromycin and 5 μg/mL blasticidin.

### 4.3 RNA extraction and reverse transcription–quantitative real‐time PCR (RT‐qPCR)

Total RNA was isolated using TRIzol reagent (Invitrogen), followed by reverse transcription using RevertAid First Strand cDNA Synthesis Kit (Thermo Scientific). Subsequently, quantitative PCR (qPCR) was conducted using SYBR™ Select Master Mix (Applied Biosystems). GAPDH was employed as endogenous control, and the relative expressions of both mRNAs and lncRNAs were determined utilizing the 2^−ΔΔCt^ method within the qPCR analysis. The specific primers for individual genes can be found in Table S2.

### 4.4 Western blotting analysis

Total cellular proteins were lysed by RIPA lysis buffer (CST). The lysates were assessed for protein content using the bicinchoninic acid method. The extraction method for conditioned medium proteins was performed as previously described.^46^ Equal amounts of proteins were loaded, performed sodium dodecyl sulfate–polyacrylamide gel electrophoresis (SDS‐PAGE), and subsequently transferred onto PVDF membranes. Subsequently, the membranes were subjected to incubation with primary antibodies that specifically targeted CX3CL1 (Abcam), DHX9 (Abcam), or β‐actin (Proteintech) overnight at 4°C. Subsequently, the membranes were exposed to horseradish peroxidase (HRP)‐conjugated secondary antibodies. Finally, the protein bands were detected using the ChemiDoc™ XRS ^+^ system (Bio‐Rad). β‐actin was utilized as a loading control.

### 4.5 EdU cell proliferation assay

Cells stably expressing PVT1 and their respective control cells were seeded in a 96‐well culture plate at a density of 4 × 10^3^ cells per well. After incubating for 24 h, cell proliferation was assessed using the Cell‐Light EdU Apollo488 In Vitro Kit (RiboBio), following the manufacturer's instructions.

### 4.6 Cell growth assay

The growth capability of GBM cells was assessed using electric cell–substrate impedance sensing (ECIS). ECIS enables real‐time monitoring and quantification of nanometer‐ to micrometer‐scale morphological changes. GBM cells, at a density of 5 × 10^3^, were seeded in an ECIS culture dish (8W10E) containing 300 μL of complete DMEM medium, followed by culture at 37°C with 5% CO_2_. Continuous measurement of cell impedance was performed at a single frequency of 16,000 Hz. The obtained data were presented as correlation between normalized impedance and time.

### 4.7 RNA sequencing (RNA‐seq) and analysis

Total RNA was isolated from U251‐sg3 and U251‐sgNC cells using TRIzol reagent (Invitrogen), followed by subsequent RNA sample quality assessment. Library preparation was performed using the NEBNext® Ultra™ RNA Library Prep Kit for Illumina®. After successful library construction, the libraries were subjected to Illumina sequencing, with validation through the Agilent 2100 Bioanalyzer. Differential gene expression was determined using the criteria |log2(Fold Change)| > 1 and *p*_adj_ < 0.05. Subsequently, enrichment analysis was conducted on the identified differentially expressed genes.

The LGG and GBM datasets from TCGA were downloaded from https://portal.gdc.cancer.gov/, while the CGGA datasets were sourced from http://www.cgga.org.cn/. The correlation between genes was assessed using Pearson correlation analysis. After intersecting correlated genes from both CGGA and TCGA databases, these genes were incorporated into the enrichment analysis. The enrichment analysis was conducted using the clusterProfiler R package.^47^

### 4.8 Human chemokine assay

The culture medium derived from U251‐sgNC, sg2, and sg3 cells was analyzed for the presence of 40 chemokines using the commercial Bio‐Plex Pro™ Human Chemokine Panel 40‐Plex (Bio‐Rad Laboratories, Inc., CA, United States), following the manufacturer's instructions. A volume of 50 μL of the undiluted sample was subjected to the assay.

### 4.9 Isolation of cytoplasmic and nuclear fractions

Isolation of cytoplasmic and nuclear fractions from U87 and U251 cells was carried out using the Nucl‐Cyto‐Mem Preparation Kit (Applygen) in accordance with the manufacturer's instructions. Subsequently, RNA was extracted from both cytoplasmic and nuclear fractions, followed by RT‐qPCR analysis. The nuclear control transcript employed was MALAT1, whereas GAPDH was utilized as the cytoplasmic control transcript.

### 4.10 Stellaris RNA fluorescent in situ hybridization (FISH)

The Stellaris RNA FISH probe sets (human PVT1 with Quasar® 670 Dye and human MALAT1 with Quasar® 570 Dye) were designed and synthesized by Biosearch Technologies. U87 and U251 cells were cultured, attached to cover glass, and subsequently permeabilized using alcohol, following the established protocols of the Stellaris RNA FISH protocol. The resulting samples were subjected to imaging using a Zeiss Imager.M2 fluorescence microscope. During image processing, the acquired Z‐stack images were converted into a maximum‐intensity projection image for analysis and presentation.

### 4.11 Animal studies

Female athymic BALB/c nude mice (4–5 weeks old) were purchased from Beijing Vital River Laboratory Animal Technology Co. Subsequently, 2 × 10^6^ stable cell populations with either high or low PVT1 expression, with their respective control cells, were intracutaneously injected into the right scapular region of the nude mice, separately. After 1 month, the mice were humanely euthanized, and the tumors were excised and sectioned following fixation. Hematoxylin and eosin (H&E) staining was performed. The animal experiments were approved by the Beijing Neurosurgical Institute Laboratory Animal Welfare and Ethics Committee.

### 4.12 RNA antisense purification (RAP)

The RAP assay using biotin‐labeled RNA was conducted following the established protocol.^48^ These probes were synthesized by the Generay Biotechnology (China) and modified with biotin at their 5′ ends. The sequence of probes can be found in Table S3. U251 cells were cultured in medium containing 100 μM 4‐thiouridine (4SU) for 24 h. Subsequently, the cells were exposed to ultraviolet (UV) radiation at 365 nm with a dosage of 0.8 J/cm^2^ for cross‐linking. Following UV cross‐linking, a mixture of 5 μg probes was incubated with 120 μL of magnetic beads and cell lysate at 67°C in a thermostatic hybridization oven, rotating at 1100 rpm, for 2 h. After the incubation, protein samples were eluted and subjected to mass spectrometry analysis. Additionally, RNA samples retained during the purification process were extracted, reverse transcribed into cDNA, and their target RNA levels were assessed using qPCR. The experimental efficiency was evaluated by calculating the percentage of the target RNA relative to the input.

### 4.13 RNA pull‐down assay and western blot analysis

The pcDNA3.1(+)‐PVT1 and pcDNA3.1(−)‐PVT1 plasmids were synthesized by Generay Biotechnology (China). Subsequently, these plasmids were linearized using a single restriction endonuclease, and the linearized plasmids were employed as templates for in vitro transcription of PVT1 and its antisense RNA, respectively. Biotin‐labeled PVT1 and its antisense RNA were generated through in vitro transcription using the MEGAscript^TM^ T7 Kit (Invitrogen, USA), following instructions provided by the kit. Bio‐16‐UTP (Invitrogen) was incorporated into the in vitro transcription reaction to facilitate the labeling of PVT1.

RNA pull‐down assay with biotin‐labeled RNA was conducted following previously established protocols.^49^ In brief, whole‐cell lysates from U251 cells were incubated with 5 μg of biotinylated PVT1 at room temperature for 1 hour. Subsequently, the complexes formed were isolated using streptavidin‐coated magnetic beads (Life Technologies). Following a series of washing steps, the isolated samples were then subjected to western blot analysis.

### 4.14 RNA immunoprecipitation (RIP)

RIP experiments were conducted on U251 cells using an anti‐DHX9 antibody (Abcam) or IgG, following the guidelines of the Magna RIP™ RNA‐Binding Protein Immunoprecipitation Kit (Millipore, USA). The extracted RNA was subsequently subjected to analysis through RT‐qPCR in accordance with the manufacturer's instructions.

### 4.15 Chromatin isolation by RNA purification (ChIRP)

The ChIRP assay was performed using the Chromatin Isolation by RNA Purification (ChIRP) Kit (BersinBio™, China) in accordance with the manufacturer's instructions. Briefly, U251 cells were incubated with formaldehyde, and their DNA was then fragmented into 100–500 bp lengths. Immunoprecipitation was conducted using PVT1 probes or RMRP probes (the same as the ones used in RAP assay). After protein collection and DNA isolation, the proteins were subjected to western blot, and high‐throughput DNA sequencing and qPCR were employed to examine the DNA sequences.

### 4.16 Transwell assay for THP1 cell chemotaxis

The transwell assay was conducted using 24‐well transwell plates with 8 μm pore size (Corning). Following a 48‐h exposure of THP1 cells to 100 ng/mL of PMA (Sigma‐Aldrich), a total of 3 × 10^5^ post‐treated THP1 cells in RPMI 1640 medium were introduced into the upper chambers. In the lower chambers, 600 μL of RPMI 1640 medium containing 500 ng/mL CX3CL1 (MCE) was added as the chemotactic stimulus. After 24 hours, the cells that had migrated into the lower chambers were fixed using methanol, stained with crystal violet, and then photographed under a microscope.

### 4.17 CD14^+^ monocyte polarization experiment

CD14^+^ monocytes were cultured in a medium containing 100 μg/mL of macrophage colony‐stimulating factor (M‐CSF, PeproTech) for 7 days. The medium was refreshed every 3 days to maintain optimal conditions for cell growth and differentiation, resulting in the generation of monocyte‐derived macrophages. Subsequently, these cells were subjected to treatments using 100 ng/mL of lipopolysaccharide (LPS, MCE), 20 ng/mL of interleukin‐4 (IL‐4, PeproTech), and 200 ng/mL of CX3CL1, respectively. After 48 h, the cells were subjected to morphological assessment through microscopy. Additionally, total cellular RNA was extracted for further analysis.

### 4.18 Correlation analysis between PVT1 expression levels and proportions of infiltrating immune cells in 325 glioma patients

RNA‐seq data from a cohort of 325 glioma samples were acquired from the Chinese Glioma Genome Atlas (CGGA).^29^ The EPIC tool was employed through R programming to derive the proportions of eight immune cell types infiltrating in each sample. Subsequently, Spearman's correlation analysis was conducted to assess the relationship between the expression levels of PVT1 and the proportions of these eight immune cell types in each sample.

### 4.19 Statistical analysis

GraphPad Prism 9.0 software (GraphPad Software, USA) was employed for conducting the statistical analyses. The data are presented as the mean ± standard deviation (SD). The normality of the data was assessed using the Shapiro–Wilk method, confirming that the data followed a normal distribution. Statistical comparisons were performed using unpaired/paired Student's *t*‐test, one‐way ANOVA, or two‐way ANOVA, as depicted in the respective figures. A *p*‐value of smaller than 0.05 was deemed to indicate statistical significance.

## AUTHOR CONTRIBUTIONS

Conceptualization—LJH, HMH, and TJ. Investigation and data collection and analysis—LJH, ZW, and CL. Bioinformatics analysis—ZW and ZZ. Study supervision—HMH and TJ. Writing–original manuscript—LJH, HMH, and TJ. Manuscript review and editing—all authors.

## CONFLICT OF INTEREST STATEMENT

The authors declare that they have no competing interests.

## CONSENT FOR PUBLICATION

All authors consent to this manuscript for publication.

## Supporting information


Figure S1

Table S1

## Data Availability

All the data used for this study are presented in the paper. The datasets used and/or analyzed during the current study are available from the corresponding author upon reasonable request.
