# Supplementary material for: PVT1 promotes proliferation and macrophage immunosuppressive polarization through STAT1 and CX3CL1 regulation in glioblastoma multiforme
Source: CNS Neurosci Ther. 2024 Jan 12;30(1):e14566. doi: 10.1111/cns.14566 (PMC10805395; doi:10.1111/cns.14566)
Supplement: Supplementary file 1 — Figure S1 [file CNS-30-e14566-s002.zip › CNS_14566_Legends.docx]

**Figure S1. PVT1 exhibits high expression levels in gliomas associated with a poor prognosis**

(A). The expression of PVT1 in glioma samples from TCGA database based on histopathological subtypes. Histological subtypes with less than 5 cases are not presented in the figure. (B). The expression of PVT1 in glioma samples from TCGA database according to the 2016 WHO molecular classification. (C). The expression of PVT1 in IDH-mutant and wild-type glioma samples from TCGA database. (D) and (E). The effect of PVT1 expression on the prognosis of patients with all WHO grade gliomas and low-grade gliomas in the TCGA database.

**Figure S2. Enrichment analysis of PVT1-correlated genes**

(A). Visualization of significant GO enrichment terms of the PVT1-correlated genes. (B). Visualization of the top10 most significant KEGG pathways of the PVT1-correlated genes.
