# Supplementary figures and images for: PVT1 promotes proliferation and macrophage immunosuppressive polarization through STAT1 and CX3CL1 regulation in glioblastoma multiforme
Source: CNS Neurosci Ther. 2024 Jan 12;30(1):e14566. doi: 10.1111/cns.14566 (PMC10805395; doi:10.1111/cns.14566)

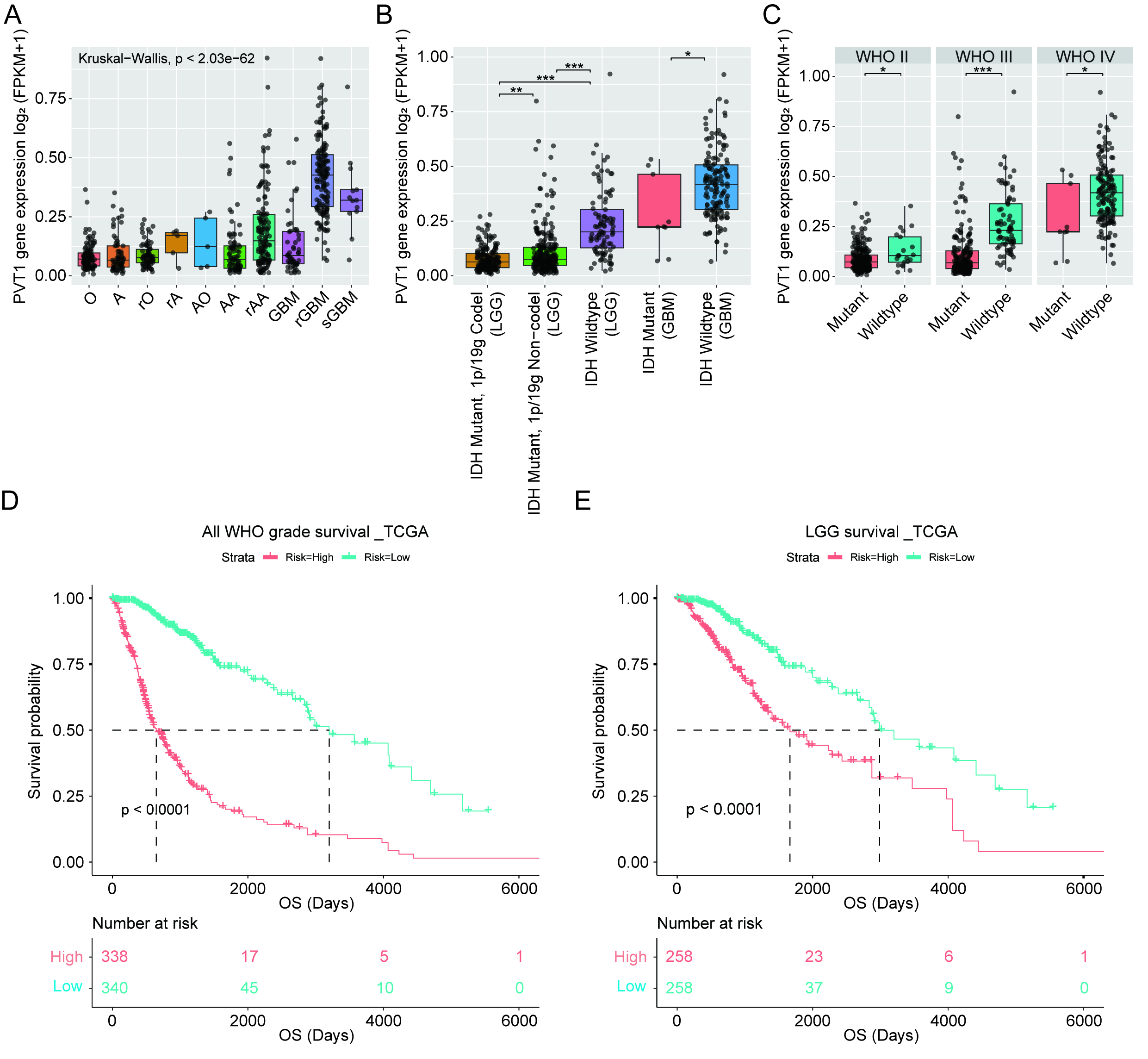

Supplement: Supplementary file 1 — Figure S1 [file CNS-30-e14566-s002.zip › cns14566-sup-0001-FigureS1.tif]

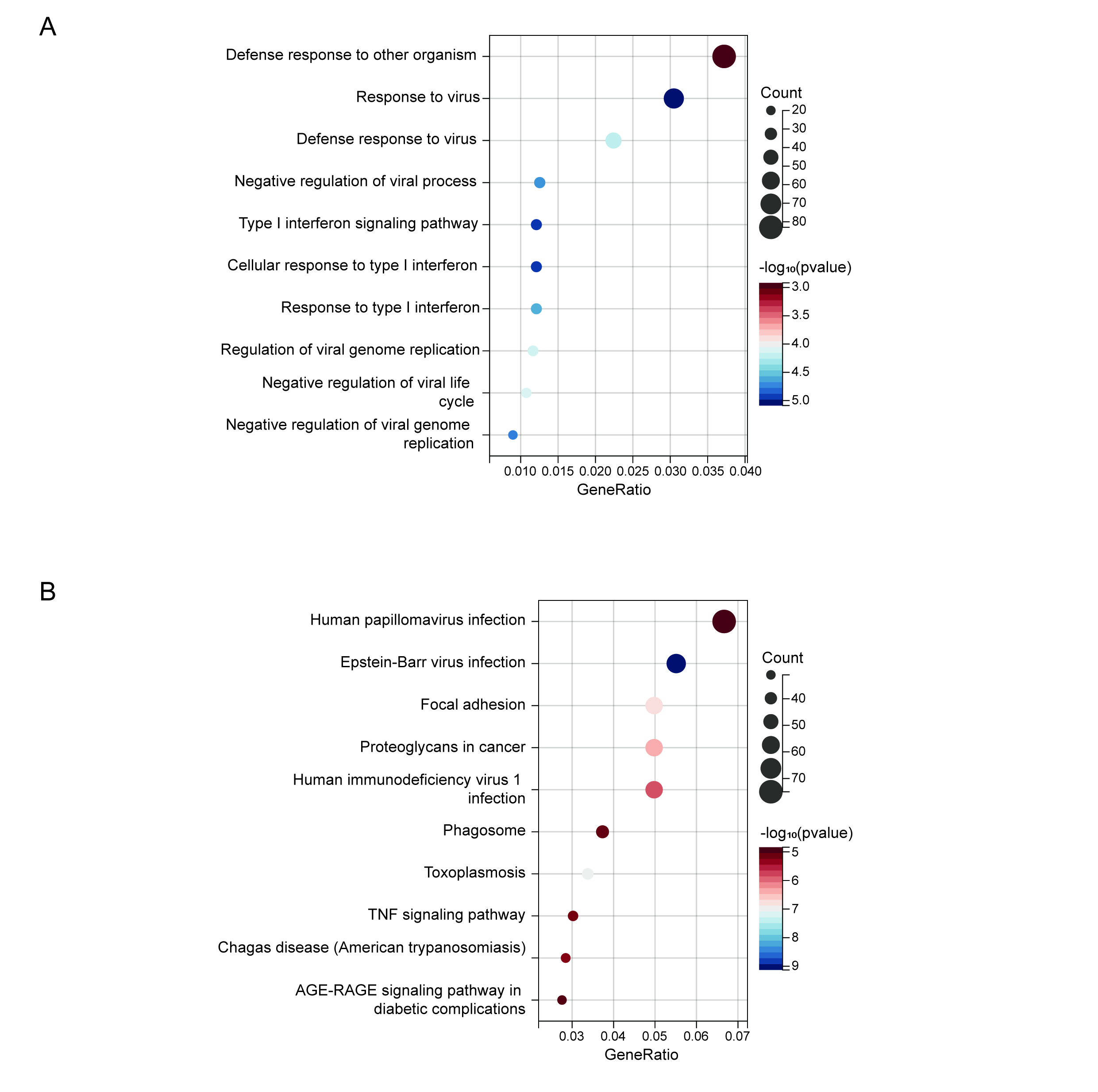

Supplement: Supplementary file 1 — Figure S1 [file CNS-30-e14566-s002.zip › cns14566-sup-0002-FigureS2.tif]
